# Assessment of non-fatal injuries among university students in Hainan: a machine learning approach to exploring key factors

**DOI:** 10.3389/fpubh.2024.1453650

**Published:** 2024-11-21

**Authors:** Kang Lu, Xiaodong Cao, Lixia Wang, Tao Huang, Lanfang Chen, Xiaodan Wang, Qiao Li

**Affiliations:** School of Public Health, Hainan Medical University, Haikou, China

**Keywords:** non-fatal injuries, university students, machine learning, Hainan Province, influencing factors

## Abstract

**Background:**

Injuries constitute a significant global public health concern, particularly among individuals aged 0–34. These injuries are affected by various social, psychological, and physiological factors and are no longer viewed merely as accidental occurrences. Existing research has identified multiple risk factors for injuries; however, they often focus on the cases of children or the older adult, neglecting the university students. Machine learning (ML) can provide advanced analytics and is better suited to complex, nonlinear data compared to traditional methods. That said, ML has been underutilized in injury research despite its great potential. To fill this gap, this study applies ML to analyze injury data among university students in Hainan Province. The purpose is to provide insights into developing effective prevention strategies. To explore the relationship between scores on the self-rating anxiety scale and self-rating depression scale and the risk of non-fatal injuries within 1 year, we categorized these scores into two groups using restricted cubic splines.

**Methods:**

Chi-square tests and LASSO regression analysis were employed to filter factors potentially associated with non-fatal injuries. The Synthetic Minority Over-Sampling Technique (SMOTE) was applied to balance the dataset. Subsequent analyses were conducted using random forest, logistic regression, decision tree, and XGBoost models. Each model underwent 10-fold cross-validation to mitigate overfitting, with hyperparameters being optimized to improve performance. SHAP was utilized to identify the primary factors influencing non-fatal injuries.

**Results:**

The Random Forest model has proved effective in this study. It identified three primary risk factors for predicting non-fatal injuries: being male, favorable household financial situation, and stable relationship. Protective factors include reduced internet time and being an only child in the family.

**Conclusion:**

The study highlighted five key factors influencing non-fatal injuries: sex, household financial situation, relationship stability, internet time, and sibling status. In identifying these factors, the Random Forest, Logistic Regression, Decision Tree, and XGBoost models demonstrated varying effectiveness, with the Random Forest model exhibiting superior performance.

## 1 Introduction

Injuries, traditionally defined as physical harm caused by the rapid transfer of energy (such as electric, thermal, or chemical energy) or by sudden hypoxia and heat loss, have now been expanded to include psychological injury, deformity, and disability (1). Injury is a worldwide public health issue that seriously threatens human health and has become the primary “killer” of people aged 0–34 in the world (2). In 2015, violence and injury prevention were included in the United Nations 2030 Agenda for Sustainable Development (3). Moreover, the Healthy China 2030 blueprint emphasizes the importance of preventing and reducing injuries (4). It was once commonly believed that injuries are accidental, unpredictable, and unavoidable. However, this perspective has changed. While the injury occurs suddenly, its causes are both external and internal, which means effective control measures can be implemented targeting the two aspects (5). Over 50 years of public health research have clarified that injuries are not accidental; there are established risk factors that can be predicted and prevented (6). Research indicates that injuries result from the complex interaction of social, psychological, and physiological factors (7). The World Health Organization reports that approximately 500,000 people die each year from violence and injuries in Europe, accounting for more than 5% of all deaths in the region. This equates to one person dying per minute (6, 8). Injuries not only cause deaths but also result in substantial socioeconomic costs. For instance, injuries cost the United States an estimated $4.2 trillion (9), and in Canada, the median cost of injuries is $5,217 (10). Between 2000 and 2016, Ontario in Canada reported an average annual fatal injury rate of 8.7 per 100,000 people (95% CI: 7.7–9.6) (11). A multinational study of 40 countries found that injuries can cause depression, with an odds ratio (OR) of 1.72 (95% CI: 1.48–1.99) for depression among those who have suffered traffic injuries (12).

Previous studies have employed statistical methods such as *t*-tests, chi-square tests, and logistic regression to analyze factors influencing injuries and predict their occurrence (13–16). While effective in revealing specific correlations, these methods exhibit limitations in handling large datasets and nonlinear relationships. Furthermore, injury research often targets specific demographics, such as children (17) and the older adult (18), with insufficient attention given to university students who are mainly young adults. In China, injuries remain the leading cause of death among children and adolescents, surpassing other disease categories (19). University students, in particular, are vulnerable to injuries due to the pressures of academic demands, as well as the psychological and lifestyle changes they undergo during this transitional period (20, 21). College students typically experience significant psychological and lifestyle changes as they transition to college life. College students are more likely to be at risk for injury due to psychological and lifestyle changes. Specifically, many college students experience higher levels of psychological stress, which not only increases symptoms of anxiety and depression but also leads to changes in coping strategies, such as increased alcohol consumption or use of other drugs (22). These changes in behavior significantly increase the likelihood of injuries, such as unintentional injuries due to alcohol consumption, delayed reactions due to sleep deprivation, and accidents (23).

In addition, some studies have noted that deterioration in mental status is also closely linked to lifestyle choices, such as unhealthy eating habits and lack of exercise, which also increase the risk of physical injury. For example, a Finnish study found an association between lifestyle behaviors, injuries, and psychological distress among college students (24). These findings suggest that college students are in a particular life stage that makes them more susceptible to psychological distress and poor lifestyle habits, which increases the risk of injury. This demographic faces distinct risks and challenges arising from their unique behaviors and living conditions, yet relevant research is still lacking.

Machine learning (ML) methods have gained considerable traction within the field of healthcare analysis, with wide applications in the research of diseases and those with large databases (25, 26). For instance, Sun et al. (27) used ML algorithms (logistic regression, XGBoost, and Random Forest) to identify and rank risk factors affecting mammographic outcomes. Ethiopian researchers applied ML to predict anemia among children, successfully identifying significant predictors of the disease (28). This approach excels at processing complex data and identifying influencing factors that traditional analysis methods may overlook, which is critical for developing targeted preventive measures (29–31). ML can uncover more profound rules by exploring data, capturing and managing multi-level and interactive nonlinear relationships between variables, thereby constructing corresponding models. Machine learning algorithms are designated to making accurate predictions, whereas traditional statistical methods only infer relationships between variables (32). Unlike traditional statistical methods, ML algorithms are data-driven and not constrained by prior assumptions (33). Given its advantages, ML has consistently outperformed traditional methods in disease prediction within the healthcare field. As a main subset of artificial intelligence, ML is a data analysis method capable of capturing the correlation between complex data. This renders it a sought-after method in risk forecasting (34, 35). ML models can greatly boost the efforts of injury prevention researchers, practitioners, and policymakers by increasing the efficiency of data collection and analysis (36). Analyzing injury-related data is challenging due to its nonlinear nature and the imbalance and complexity of outcome variables. ML outperforms traditional methods in handling unbalanced data (37). Furthermore, ML is useful for feature selection. Despite its extensive application to predict suicidal behavior and explore the influencing factors (38), rarely have there been studies using this method to examine the influencing factors and prediction of injury.

Hainan is China’s only tropical island province, characterized by high temperatures and high humidity (39). This climate makes outdoor activities and water sports more prevalent among university students. It may also increase the risk of injuries due to heat stroke, dehydration, and sports-related falls or abrasions. In addition, frequent rainfall and typhoons may cause slippery road surfaces, which may increase the incidence of unintentional injuries. In addition, medical resources are limited in some areas of Hainan Province, which may affect timely treatment after an injury, thus exacerbating the severity of the injury (40). These geographic and environmental factors may play a vital role in the incidence of nonfatal injuries among college students.

To address this research gap and enhance the accuracy and efficiency of the injury prediction model, we employed ML methods to analyze injury data among university students in Hainan Province, exploring factors closely associated with injury occurrence. The findings are expected to provide robust scientific support for developing effective prevention strategies.

## 2 Materials and methods

### 2.1 Research population

The study population involves undergraduate students enrolled in higher education institutions in Hainan Province.

### 2.2 Inclusion and exclusion criteria

#### 2.2.1 Inclusion criteria

The subjects who met the following criteria were included in the study:

1. Current undergraduate students at higher education institutions in Hainan Province.
2. Students capable of understanding and independently completing the questionnaire.

#### 2.2.2 Exclusion criteria

Subjects were excluded if they:

1. Were unable to understand or independently complete the questionnaire, including those with cognitive disabilities or reading comprehension disorders.
2. Did not provide valid consent to participate in this study.

### 2.3 Sampling methods

This study employs multistage random sampling to ensure comprehensive coverage and representativeness of samples from 11 higher education institutions in Hainan Province. The sampling process included three stages:

*Stage 1: institution selection*.

A list of 11 higher education institutions in Hainan Province was compiled, each assigned a unique code. Subsequently, three institutions were selected using computer-generated random numbers through simple random sampling to ensure equal selection probability.

*Stage 2: class selection*.

Within each selected institution, clusters of grades were formed using whole cluster sampling. Three classes were randomly chosen from each grade level using the same random sampling method. Specifically, the sample included all undergraduate college grades (freshman through senior year, including the fifth year for medical majors) to ensure coverage of students at different stages of study and to avoid selection bias.

*Stage 3: student selection.*

All students in the selected classes were included as survey respondents. In the end, we got data for 3,128 students.

### 2.4 Quality control

To ensure data accuracy and reliability, the following quality control measures were conducted:

1. Investigator training: guided by the project leader, all survey personnel received standardized training on survey methods, techniques, and bias control. This was to ensure proficiency and adherence to protocols.
2. Pre-survey implementation: a subset of the target population was selected for a pre-survey to evaluate the questionnaire’s reliability and validity. Data from the pre-survey were analyzed for inconsistencies and logical flaws, facilitating necessary adjustments accordingly.
3. Data quality monitoring: THE team regularly assessed data accuracy and completeness during data collection, thereby promptly identifying and rectifying any irregularities. A dual data entry system was employed, with two clerks independently entering the same data to identify and correct errors.
4. Logical review of the questionnaire: a comprehensive review was conducted to ensure all questions were coherent and executable before finalizing the questionnaire design. This included verifying question sequence, response choice completeness, and estimated completion time.
5. Ethical considerations: participation was voluntary, with no incentives or penalties. The process adheres to principles of ethical research and was under informed consent of the participants.
6. Non-response bias control: we used face-to-face or distributed questionnaires in this study. We collected the questionnaires through lecturers or tutors, through which we minimized the non-response bias.
7. Protection of data privacy: in this study, all participants’ data were de-identified at the time of collection. Personally identifiable information, such as the participant’s name and school number, were not collected but were replaced with unique numbers to ensure anonymity. All data were accessible only to authorized members of the research team. In addition, strict confidentiality protocols were adopted to ensure that all sensitive information (e.g., mental health and family status) was appropriately protected throughout the study. Upon completion of the study, the data will be retained for 10 years for subsequent research, after which it will be securely destroyed.

These measures were implemented to reduce bias and enhance the reliability and validity of the results through rigorous quality control.

### 2.5 Research instruments

#### 2.5.1 Demographic information

The research team designed a demographic information section to collect the general characteristics of undergraduate students in Hainan Province for comprehensive analysis. It includes basic demographic information, family background, and school living conditions of the participants. The questionnaire encompasses a range of socio-demographic variables, including sex, grade, major, hometown, physical health, sleep quality, likes sports, personality traits, likes adventure, and internet time. Such a design aims to assess the respondents’ socio-demographic situation. Additionally, the questionnaire delves into the respondents’ family background, including their parental relationships, parenting style, and family financial status. It also inquires about their relationship with their classmates, academic pressure, bedroom environment, and their relationship stability to evaluate their on-campus psychological conditions.

#### 2.5.2 The Zung self-rating anxiety scale (SAS)

The study utilized SAS, a self-report scale consisting of 20 items that assess a wide range of anxiety symptoms, both psychological (e.g., fear, nervousness) and somatic (e.g., trembling, accelerated heartbeat). Each item was rated on a 4-point Likert scale, with responses ranging from 1 (none or very little of the time) to 4 (most or all of the time). Participants were asked to base their responses on their experiences over the past week. The items included both negative and positive experiences, with the latter being reverse scored. The scores for the 20 items were summed to yield a raw score, which was then multiplied by 1.25 to produce a total score (25–100) (41). The SAS has demonstrated satisfactory psychometric properties, with a Cronbach’s alpha value of 0.82 (42).

#### 2.5.3 The Zung self-rating depression scale (SDS)

SDS was employed to assess the depression of the respondents. The 20-item scale evaluates the mood symptoms of participants over the past week. Each item was scored on a 4-point Likert scale based on the frequency of symptoms, with responses ranging from 1 (none or very little of the time) to 4 (the vast majority or all of the time). The score for each item was calculated to obtain a raw score, and the standardized score was equal to the raw score multiplied by 1.25. The Chinese version of the questionnaire has been widely used in previous studies and has demonstrated favorable reliability and validity (43–45). The anxiety and depressive symptoms are often characterized by long-term persistence, and symptoms may persist for years with only limited improvement even after treatment. A six-year prospective study found that significant reductions in anxiety and depressive symptoms were often challenging to achieve over long periods (46). Based on this premise, we hypothesized in the present study that recent psychological status would be a valid reflection of emotional state over the last year.

#### 2.5.4 Conditions of non-fatal injuries

The questionnaire included a section to assess the prevalence of non-fatal injuries sustained in the past 12 months. Injuries were defined as those diagnosed by a medical professional or resulting in a period of leave (from school, work, or rest) exceeding 1 day. Respondents answered “yes” or “no” to the questions. A “yes” response indicates the occurrence of an injury within the past year.

#### 2.5.5 Sleep quality

The literature states that poor sleep quality leads to decreased emotion regulation and affects college students’ interpersonal functioning through increased impulsive behaviors (47). Additionally, there is a significant relationship between sleep quality and anxiety levels, thereby increasing the risk of injury in response to high-stress situations (48).

#### 2.5.6 Personality traits and tendencies for adventure

Extraverts typically exhibit higher social demands and activity levels and are likelier to engage in high-risk activities such as extreme sports. Participation in such activities is not only associated with thrill-seeking but may also increase the probability of injury. For example, one study showed that extraversion and openness personality traits were significant predictors of extreme sports participation and injury risk (49). Additionally, research has shown that there is also an association between extraversion and adventure-related risk behaviors, which further increases the likelihood that extroverted individuals will experience injuries or negative consequences (50). Therefore, we focused on analyzing the impact of these variables on nonfatal injuries among college students in this study.

### 2.6 Statistical methods

This study employs the R software (version 4.0.3) for data analysis, with all work conducted in the RStudio environment. The primary R packages used for data processing and analysis included tidyverse, mlr3verse, glmnet, rms, and iml. Specifically, tidyverse was used for data wrangling; mlr3verse provided tools related to ML; glmnet implemented Lasso regression; the rms package was used to construct restricted cubic spline regression; and the iml package interpreted and visualized the output of ML models. These tools ensured analytical rigor and result reliability. To investigate the correlation between SAS and SDS scores and the risk of non-fatal injuries within 1 year, we categorized both scores into two groups using restricted cubic splines for analysis. The chi-square test and Lasso regression were used to screen variables associated with student injuries, thus simplifying the model and enhancing prediction accuracy. The screened data were divided into a training set and a test set. The training set was balanced using the Synthetic Minority Over-sampling Technique (SMOTE) (51). Four classification models were constructed using 10-fold cross-validation techniques: Random Forest, Decision Tree, Logistic Regression, and XGBoost. In this study, we used F1 and AUC values as evaluation indicators to compare the performance of each model. The F1 value is the harmonic mean of the model’s precision and recall, making it particularly suitable for class-imbalanced data sets. The AUC value measures a model’s ability to distinguish between positive and negative samples, serving as a comprehensive indicator for evaluating classification models. Combining these two indicators helps gain a comprehensive understanding of each model’s strengths and weaknesses. This offers a scientific basis for model selection. In this study, the dependent variable is the extent of non-fatal injuries, while the independent variables include sociodemographic factors, family circumstances, and school conditions. The significance level for univariate analysis was set at *α* = 0.05.

### 2.7 Model interpretation

Shapley Additive exPlanations (SHAP) is an advanced interpretation method derived from cooperative game theory that aims to rationally assign the output of a machine learning model to individual input features by calculating Shapley values (52). This technique ensures a fair assessment of each feature’s impact by calculating the average incremental contribution of each feature across all possible combinations of features, thus providing a reliable and accurate interpretation of model predictions. The introduction of SHAP into predictive modeling can offer valuable insights into the influence of individual features on predicted outcomes, helping to enhance the transparency and interpretability of complex models, especially in critical decision-making scenarios.

Unlike traditional explanatory methods, SHAP not only measures feature importance but also offers a more nuanced perspective that reveals the specific relationship between features and predicted outcomes, thus addressing the limitations of traditional approaches. SHA *p-*values are computed individually for each feature in every prediction sample, quantifying the positive or negative impact of each feature on the final prediction. Through this approach, SHAP provides deeper insights that help us better understand the model’s decision-making process.

### 2.8 Flow chart

**Figure 1:**
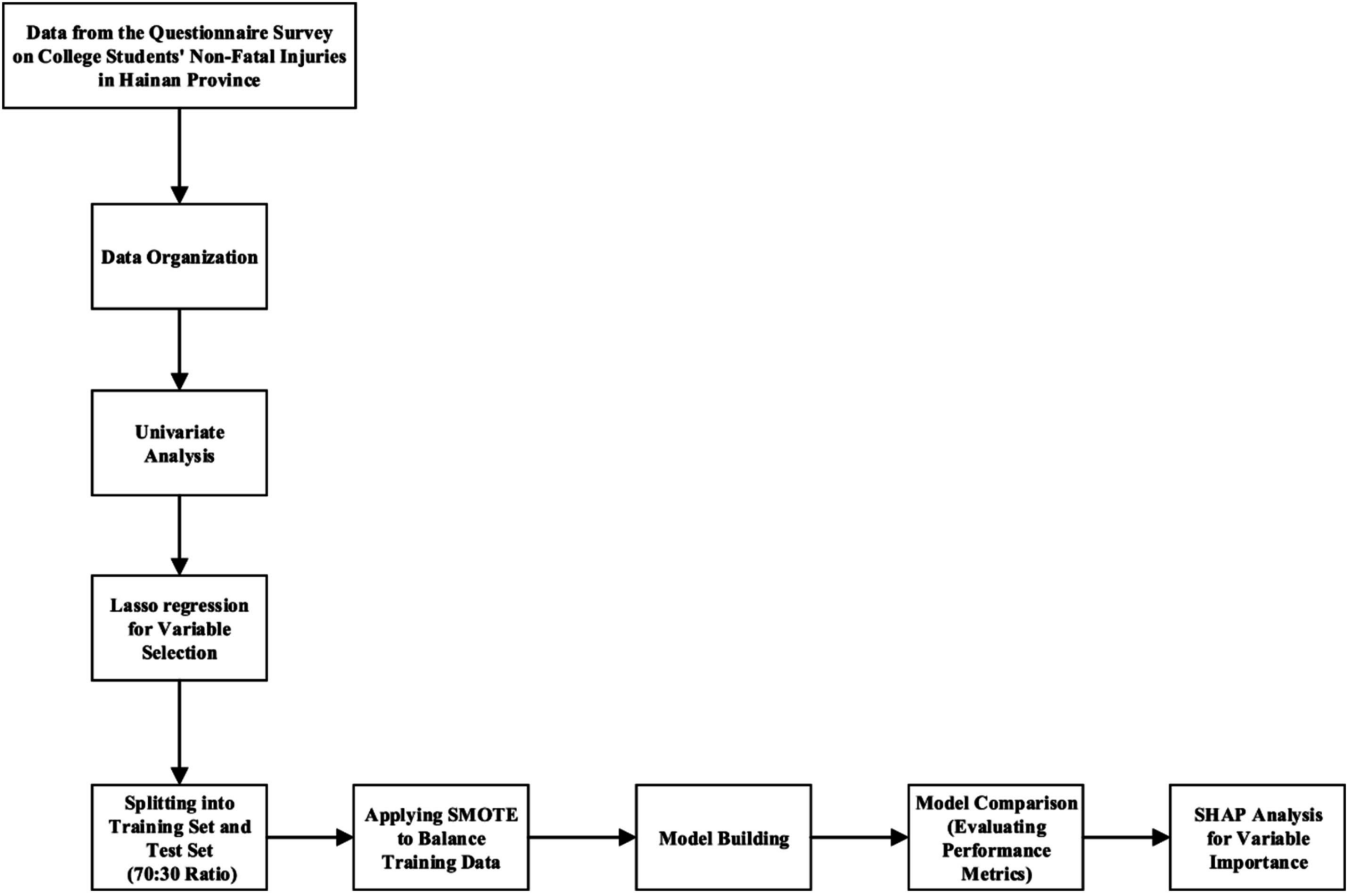
Statistical analysis and machine learning workflow diagram.

The thesis analysis process is shown in Figure 1.

### 2.9 Ethical approval

The Ethical Committee of Hainan Medical College has approved the survey in this study (HYLL-2023-104).

## 3 Results

### 3.1 General demographic characteristics

The survey was completed in 2021, and the analysis was conducted in 2023. Out of 3,134 distributed questionnaires, 3,128 are valid, yielding a validity rate of 99.8%. The respondents comprised 1,432 males (45.8%) and 1,696 females (54.2%). Regarding their majors, 914 respondents (29.2%) were from the humanities and social sciences, 1,308 (41.8%) from science and engineering, 597 (19.1%) from medical sciences, and 309 (9.9%) studied other disciplines. A total of 430 individuals (13.7%) reported experiencing non-fatal injuries in the past 12 months before they participated in the survey.

### 3.2 Association between psychological symptoms and non-fatal injury risk within 1 year

**Figure 2:**
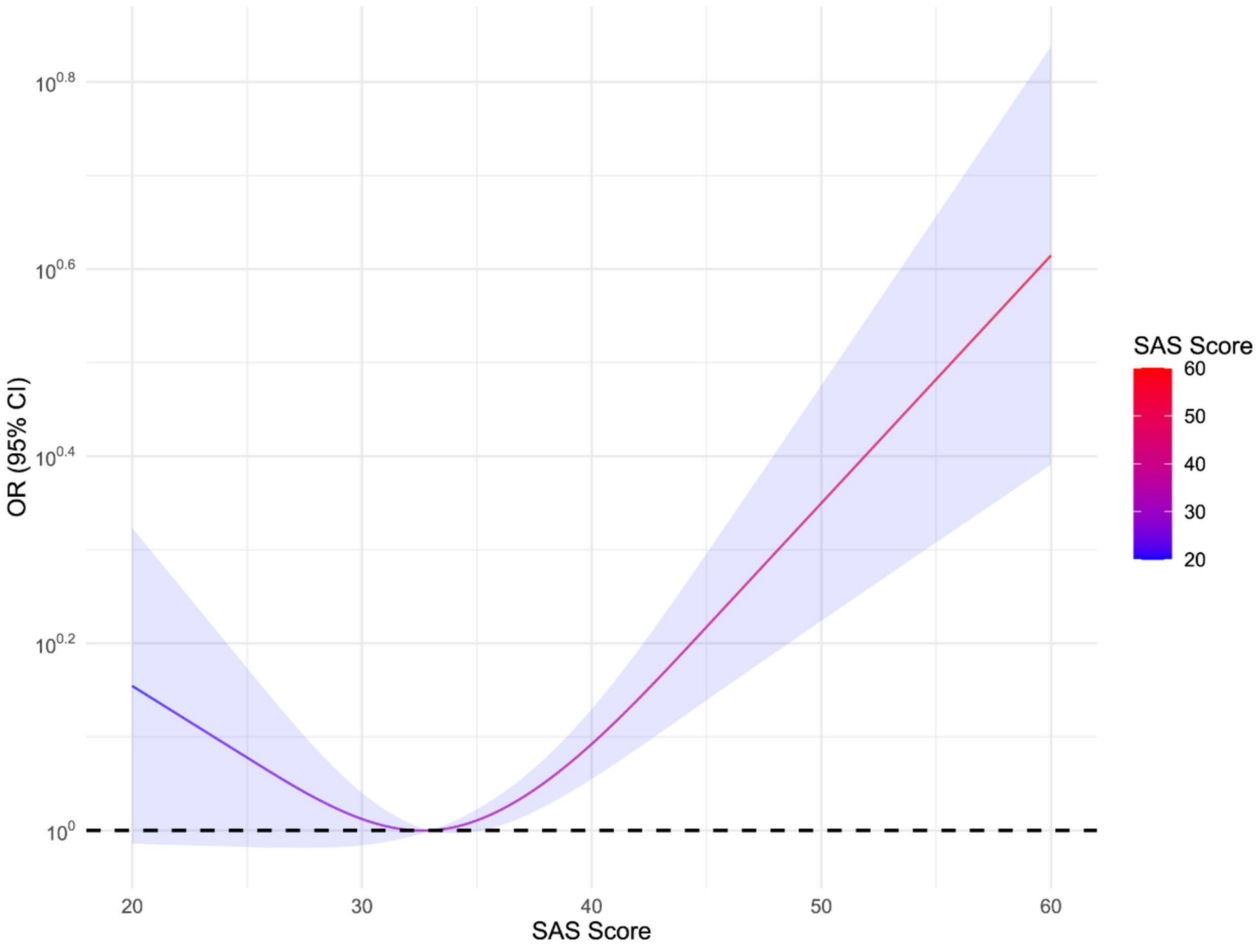
Correlation between SAS score and risk of non-fatal injuries within 1 year.

Figure 2 illustrates the correlation between SAS scores and the risk of non-fatal injury within 1 year. The horizontal axis represents the SAS score, where a higher score indicates more severe anxiety symptoms. The vertical axis represents the OR value. The curve suggests an association between higher SAS scores and an increased risk of non-fatal injury. However, this relationship is not statistically significant due to the wide confidence intervals at higher scores, indicating increased uncertainty. To facilitate analysis, SAS scores were divided into two groups: low-to-moderate anxiety (33 points and below) and high anxiety (above 33 points).

**Figure 3:**
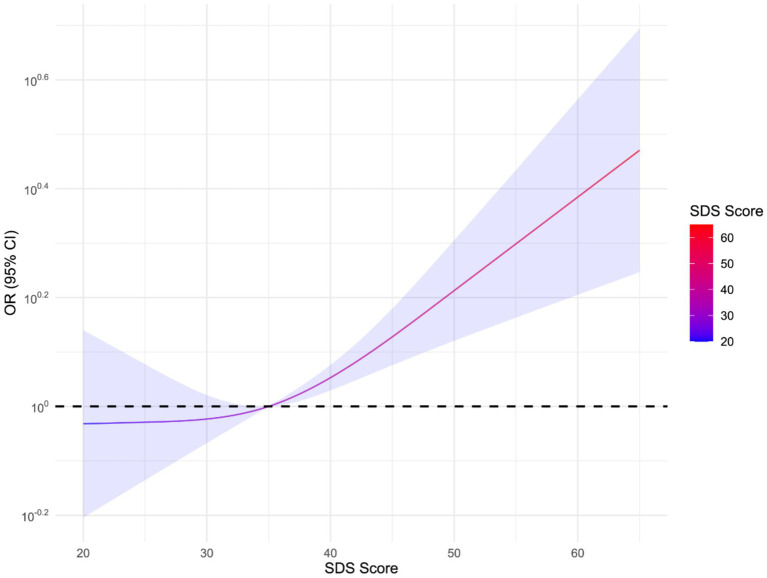
Correlation between SDS score and risk of non-fatal injuries within 1 year.

Figure 3 depicts the correlation between SDS scores and the risk of non-fatal injury within 1 year. The horizontal axis represents the SDS score, with higher scores indicating more severe depressive symptoms. The curve demonstrates a significant increase in OR values as SDS scores rise. At an SDS score of 35, the OR value is 1 (indicated by the dashed line). This suggests no significant association between depression and non-fatal injury risk at this point. However, when the scores exceed 35, there exhibits a positive correlation between depression scores and the risk of non-fatal injury, with the risk increasing significantly. To facilitate analysis, SDS scores were divided into two groups: low-to-moderate depression (35 points and below) and high depression (above 35 points).

### 3.3 Univariate analysis of non-fatal injuries cases among university students in Hainan Province

The chi-square test reveals a statistically significant association between variables and the risk of non-fatal injuries (see Table 1). Statistically, significant differences are observed across different grades, sexes, only child status, physical health statuses, relationships with classmates, levels of academic pressure, bedroom environments, personality traits, tendencies for adventure, household financial situations, parenting styles, relationship conditions, internet usage, and depressive conditions.

**Table 1:** Factors affecting the risk of non-fatal injuries among university students in Hainan Province (chi-square test).

	Non-fatal injuries		Statistical value	*P*-value
	Yes (*N* = 430)	No (*N* = 2,698)		
Grade			10.37	0.016
Freshman	91 (11.9%)	676 (88.1%)		
Sophomore	149 (16.8%)	736 (83.2%)		
Junior	106 (12.8%)	720 (87.2%)		
Senior and above	84 (12.9%)	566 (87.1%)		
Major			7.21	0.066
Humanities and Social Sciences	122 (13.3%)	792 (86.7%)		
Science and Engineering	198 (15.1%)	1,110 (84.9%)		
Medical Sciences	64 (10.7%)	533 (89.3%)		
Other	46 (14.9%)	263 (85.1%)		
Sex			18.84	<0.001
Male	239 (16.7%)	1,193 (83.3%)		
Female	191 (11.3%)	1,505 (88.7%)		
Hometown			6.12	0.106
City	140 (12.6%)	967 (87.4%)		
Town	150 (15.9%)	793 (84.1%)		
Township	71 (13.9%)	438 (86.1%)		
Rural area	69 (12.1%)	500 (87.9%)		
Only child			18.20	**<0.001**
Yes	216 (17.0%)	1,058 (83.0%)		
No	214 (11.5%)	1,640 (88.5%)		
Physical health			24.94	**<0.001**
Very Good	228 (13.2%)	1,493 (86.8%)		
Average	174 (13.2%)	1,145 (86.8%)		
Poor	28 (31.8%)	60 (68.2%)		
Relationships with Classmates			9.13	**0.010**
Good or very good	313 (13.1%)	2082 (86.9%)		
Average	99 (15.0%)	561 (85.0%)		
Poor or very poor	18 (24.7%)	55 (75.3%)		
Academic pressure			14.68	**<0.001**
High	113 (16.1%)	588 (83.9%)		
Moderate	255 (12.2%)	1841 (87.8%)		
None	62 (18.7%)	269 (81.3%)		
Bedroom environment			5.40	**0.020**
Quiet	208 (12.4%)	1,471 (87.6%)		
Noisy	222 (15.3%)	1,227 (84.7%)		
Sleep quality			3.30	0.192
Good or very good	273 (13.7%)	1725 (86.3%)		
Average	118 (13.0%)	791 (87.0%)		
Poor or very poor	39 (17.6%)	182 (82.4%)		
Likes sports			0.66	0.417
Yes	248 (14.2%)	1,496 (85.8%)		
No	182 (13.2%)	1,202 (86.8%)		
Personality traits			5.10	**0.024**
Extroverted	256 (15.0%)	1,445 (85.0%)		
Introverted	174 (12.2%)	1,253 (87.8%)		
Likes adventure			4.92	**0.027**
Yes	247 (15.1%)	1,391 (84.9%)		
No	183 (12.3%)	1,307 (87.7%)		
Household financial situation			19.05	**<0.001**
Good	113 (15.8%)	602 (84.2%)		
Moderate	245 (12.0%)	1803 (88.0%)		
Poor	72 (19.7%)	293 (80.3%)		
Parental relations			3.29	0.193
Very Good	241 (13.2%)	1,585 (86.8%)		
Average	163 (14.0%)	999 (86.0%)		
Estranged	26 (18.6%)	114 (81.4%)		
Parenting style			14.47	**0.002**
Authoritarian	55 (12.9%)	372 (87.1%)		
Authoritative	221 (12.1%)	1,598 (87.9%)		
Permissive	85 (17.3%)	407 (82.7%)		
Neglectful	69 (17.7%)	321 (82.3%)		
Relationship going well			11.82	**0.003**
Smoothly	169 (16.4%)	861 (83.6%)		
Not smoothly	112 (14.0%)	688 (86.0%)		
Never been in a relationship	149 (11.5%)	1,149 (88.5%)		
Internet time			9.19	**0.002**
6 h or less	238 (12.3%)	1703 (87.7%)		
More than 6 h	192 (16.2%)	995 (83.8%)		
Anxiety conditions			0.21	0.645
High anxiety group	229 (14.0%)	1,401 (86.0%)		
Low-to-moderate anxiety group	201 (13.4%)	1,297 (86.6%)		
Depressive conditions			6.35	**0.012**
High depression group	254 (15.2%)	1,414 (84.8%)		
Low-to-moderate depression group	176 (12.1%)	1,284 (87.9%)		

*P-values with bolded numbers are <0.05.*

Significant differences were observed across several variables (see Table 2). For Grade, distinctions were found between Freshman and Sophomore students, Sophomore and Junior students, and Sophomore and “Senior and above” students. In Physical Health, marked differences were identified between Very Good and Poor as well as between Average and Poor. For Relationships with Classmates, significant differences were noted between Good or Very Good and Poor or Very Poor and between Average and Poor or Very Poor. Academic Pressure revealed differences between High and Moderate levels and between Moderate and None. Household Financial Situation comparisons showed differences between Good and Moderate and between Moderate and Poor. In Parenting Style, significant contrasts were found between Authoritative and Permissive and between Authoritative and Neglectful. Finally, Relationship Going well displayed a notable difference between Smoothly and Never been in a relationship.

**Table 2:** Chi-square partition analysis for variables with multiple categories.

Comparison	Statistical value	*P*-value
Grade		
Freshman and sophomore	8.18	**0.004**
Freshman and junior	0.34	0.557
Freshman and “senior and above”	0.36	0.546
Sophomore and junior	5.40	**0.020**
Sophomore and “senior and above”	4.46	**0.035**
Junior and “senior and above”	3.0 × 10^−3^	0.959
Physical health		
Very good and average	2.0 × 10^−3^	0.964
Very good and poor	23.76	**<0.001**
Average and poor	23.28	**<0.001**
Relationships with classmates		
Good or very good and average	1.65	0.198
Good or very good and poor or very poor	8.19	**0.004**
Average and poor or very poor	4.57	**0.033**
Academic pressure		
High and moderate	7.19	**0.007**
High and none	1.09	0.297
Moderate and none	10.85	**0.001**
Household financial situation		
Good and moderate	6.93	**0.008**
Good and poor	2.62	0.106
Moderate and poor	16.37	**<0.001**
Parenting style		
Authoritarian and authoritative	0.17	0.679
Authoritarian and permissive	3.42	0.064
Authoritarian and neglectful	3.67	0.056
Authoritative and permissive	8.86	**0.003**
Authoritative and neglectful	8.65	**0.003**
Permissive and neglectful	0.03	0.872
Relationship going well		
Smoothly and not smoothly	2.01	0.156
Smoothly and never been in a relationship	11.83	**0.001**
Not smoothly and never been in a relationship	2.89	0.089

Extremely small Chi-square statistics are shown in scientific notation (e.g., 3.0 × 10^−3^) for clarity and to maintain two decimal place formatting. *P*-values with bolded numbers are <0.05.

### 3.4 Variable selection for non-fatal injuries prediction using machine learning

**Figure 4:**
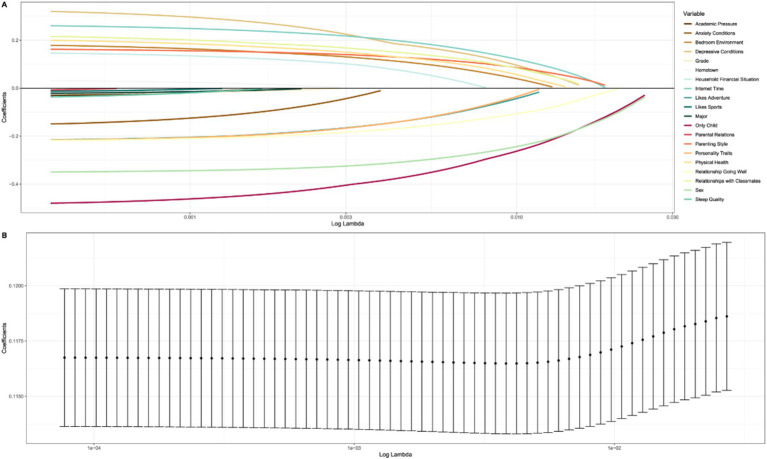
Lasso selection pathway for non-fatal injuries risk factors among university students in Hainan Province. **(A)** Coefficient profiles of risk factors as a function of the regularization parameter (Log Lambda). **(B)** Cross-validation results showing the mean square error for different values of Log Lambda.

We employed the LASSO regression method to screen out variables significantly associated with non-fatal injuries. Initially, a series of potential predictor variables were considered, including grade, major, sex, and 20 variables. The LASSO path diagram (Figure 4A) illustrates that as the regularization coefficient increases, the coefficients of many predictors gradually decrease to zero. By selecting lambda.1se (Figure 4B) and adjusting the regularization parameter *λ*, we effectively identified and excluded variables contributing the least to the model predictions, as their coefficients collapsed to zero. Specifically, the LASSO regression model identified “grade,” “major,” “hometown,” “academic pressure,” “sleep quality,” “likes sports,” and “parental relations” as variables with a negligible impact on the prediction results, leading to their exclusion. In addition, based on the chi-square test results, the variable “anxiety conditions” was excluded. Ultimately, 12 variables with significant predictive power were retained to construct a model to optimize prediction accuracy.

### 3.5 Construction of machine learning models

**Figure 5:**
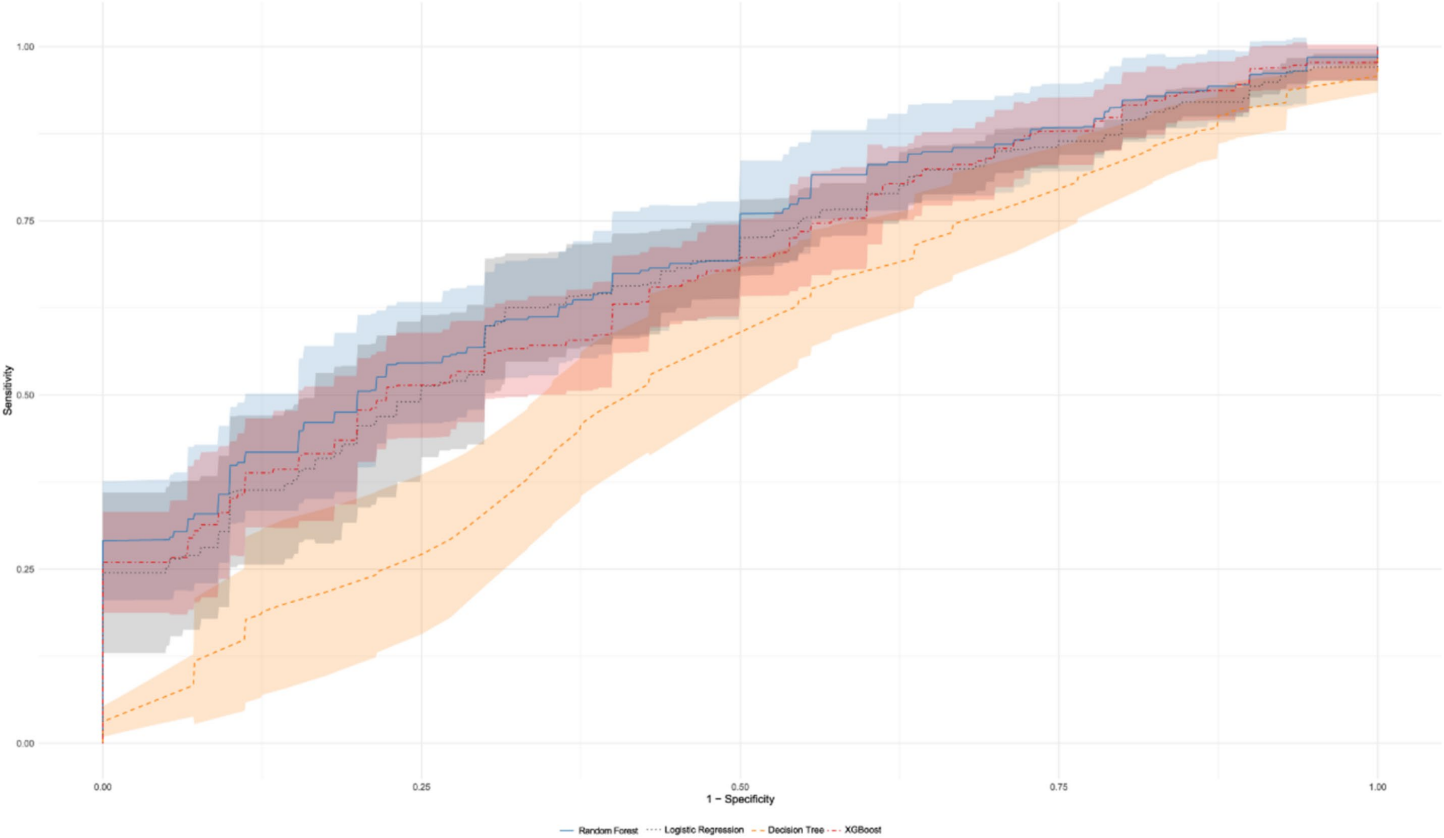
ROC curve comparison with confidence intervals for different models. This figure displays the ROC curves and their confidence intervals for the random forest, logistic regression, decision tree, and XGBoost models. The shaded region represents the confidence intervals for each model.

Based on the model performance indicators (AUC and F1 score) presented in Table 3, along with the ROC curve in Figure 5, the performance of different models in predicting non-fatal injuries was evaluated. The Random Forest model demonstrated the best overall prediction performance, as it achieved the highest AUC value of 0.702 and an F1 score of 0.925, which indicates superior accuracy in distinguishing positive and negative samples. The XGBoost model achieved an AUC value of 0.688 and an F1 score of 0.926, while the Logistic Regression model had an AUC value of 0.675 and an F1 score of 0.926. Although their AUC values are slightly lower than that of Random Forest, their performance in prediction remains excellent. In contrast, the Decision Tree model had the lowest AUC value of 0.548 and an F1 score of 0.898, reflecting its poorer ability to distinguish between positive and negative samples and relatively weak overall prediction performance. Therefore, the Random Forest model is recommended as the preferred model, with XGBoost and Logistic Regression considered effective alternatives; whereas the Decision Tree model is not recommended as the primary prediction tool. Consequently, we choose the Random Forest model to analyze the influencing factors of non-fatal injuries.

**Table 3:** Performance metrics comparison of models.

Model	AUC	F1
Random forest	0.702	0.925
XGBoost	0.688	0.926
Decision tree	0.548	0.898
Logistic regression	0.675	0.926

**Figure 6:**
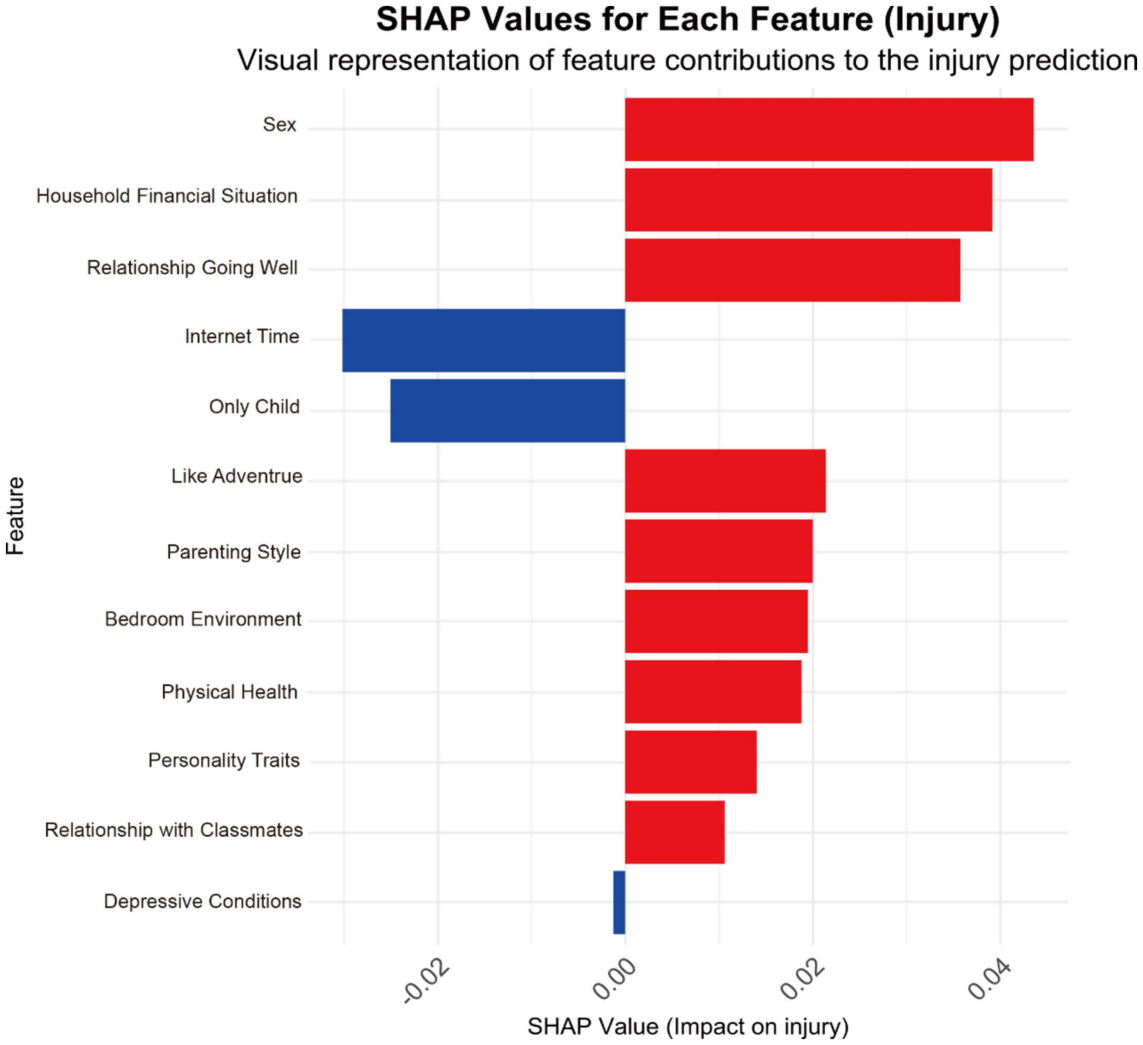
SHAP values for each feature (non-fatal injuries). Red bars represent positive impacts, and blue bars represent negative impacts. Sex = Male, Relationships with Classmates = Good or Very Good, Relationship Going Well = Smoothly, Physical Health = Very Good, Personality Traits = Extroverted, Parenting Style = Authoritative, Only Child = Yes, Likes Adventure = Yes, Internet Time = 6 h or less, Household Financial Situation = Good, Depressive Conditions = Low-to-moderate depression group, Bedroom Environment = Quiet.

Based on the Random Forest model’s performance, we calculated each feature’s importance according to the absolute SHAP value, with blue and red indicating the negative and positive contribution of the feature, respectively. Higher SHAP values on the chart indicate that the feature has a more significant impact on the model’s predictions and a higher predicted risk of nonfatal injuries. Conversely, a lower SHAP value indicates a lesser influence on the prediction and a lower risk of prediction. Figure 6 clearly shows the contribution of the top 12 contributing features to model predictions, simplifying the interpretation of complex model outputs and deepening the understanding of the relationship between features and results. It is worth noting that gender was the most critical predictor in this study, followed by family finances.

Specifically, the analysis identifies “sex” as the most critical factor, with males showing a higher probability of non-fatal injuries. Individuals with good family financial situations also exhibit a higher risk of injury. Those in smooth relationship conditions similarly show a higher risk of injury. Individuals who enjoy adventures show a higher probability of non-fatal injuries. Among different parenting styles, authoritative parenting is associated with a higher probability of non-fatal injuries, and so is the influence of noisy bedroom environments. Moreover, extroverted individuals and those with very good physical health have a higher incidence of non-fatal injuries. Students with good or excellent relationships with classmates also show a higher risk of non-fatal injuries. Conversely, less internet time (6 h or less) is associated with a lower incidence of non-fatal injuries. The incidence of non-fatal injuries is lower among only children in the college student population. The impact of depression on the prediction of non-fatal injuries is minimal and almost negligible.

## 4 Discussion

### 4.1 Incidence and regional comparison of non-fatal injuries

In this study that targets students at Hainan University, the incidence of non-fatal injuries was found to be 13.7%. This rate is notably lower than the incidence reported in other studies focusing on university students (53, 54). For instance, the incidence rate of unintentional injuries among students in 50 colleges and universities in China was 47.9% (20). Since the sample of this study focused on Hainan Province, regional and cultural factors specific to Hainan may play a role in the results. Hainan’s tropical climate may lead students to engage in more outdoor activities, increasing the risk of physical activity-related injuries (20). Parenting styles are similar to those in the rest of China because college students in Hainan Province come from all over the country. Thus, however, climatic characteristics may limit the generalizability of the findings, and future studies should consider validating these results in a broader geographic and cultural context.

Regional factors in Hainan Province may play a key role in reducing the incidence of non-fatal injuries. Socio-cultural and infrastructural differences may have an impact on injury rates. According to the literature (82), Hainan has a slightly lower level of education and socioeconomic development compared to other developed coastal provinces, which leads to lower participation in high-risk behaviors (e.g., risky driving or vigorous sports) among the local population, thus reducing the overall incidence of non-fatal injuries. The level of regional health development and socioeconomic status (e.g., education level, economic income) directly affects a population’s health risk level (83). Hainan has an intermediate level of socioeconomic development, and this socioeconomic background may lead to a local population that prefers a low-risk lifestyle, which in turn affects the incidence of non-fatal injuries. This suggests that regional factors may play a crucial role in this discrepancy. Therefore, further investigation into the potential contributing factors is warranted, as understanding these variations could contribute to developing strategies to reduce the occurrence of non-fatal injuries among university students.

### 4.2 Model excluded variables

During the model construction process, we chose variables such as grade level, major, hometown, sleep quality, and parental relationships because these variables have been shown in the existing literature to be closely related to college students’ health, behavioral patterns, and academic performance. For example, the literature suggests that family relationships (e.g., parental support) and hometown background (e.g., socioeconomic conditions) also play an essential role in influencing college students’ social support systems and resilience. Thus, many studies have identified these variables as critical factors in understanding college students’ mental health and academic performance (55, 56). Research suggests that personal history of mental health conditions and family dynamics (e.g., lack of family support or a history of mental illness in the family) can predispose students to mental health issues (57). Although these variables were excluded from the LASSO regression model because they contributed less to the model, they are still potentially influential from a theoretical and literature perspective regarding college students’ mental health and behavioral patterns.

Although we initially included these variables, the LASSO regression model excluded grade, major, hometown, sleep quality, and parental relationship, suggesting that they contributed less to the risk of nonfatal injuries. LASSO prevents overfitting by introducing penalty terms to reduce unimportant variables, which, although considered critical factors in the literature, may not have significantly impacted the dataset of the current study. This may be related to our particular sample and context or suggest that these variables may have a weaker role in influencing nonfatal injuries among college students. Future research could further explore the role of these variables in different populations.

### 4.3 Factors influencing non-fatal injuries

In the contemporary field of predictive analytics, ML models stand at the forefront, eclipsing traditional statistical methods by their ability to build accurate predictive models from datasets of limited size but high dimensional feature space. Despite their advanced capabilities, these models are often criticized for their lack of transparency (often referred to as the “black-box” problem), which hinders the understanding of their internal mechanisms (58). The SHAP algorithm used in this study effectively lifts this veil and improves the transparency of the model by quantifying the impact of individual features on the prediction results.

The SHAP plot based on the Random Forest algorithm identifies essential features for predicting non-fatal injuries; the first 5 features are sex, where males are prone to suffer from non-fatal injury, which is the most critical variable; individuals with favorable family financial situations have a higher risk of injury; and those in smooth and stable relationships show a higher risk of injury. Conversely, those who spend less time on the internet (6 h or less) and only children are less likely to suffer from non-fatal injuries in examining nonfatal injuries among university students, this study has revealed a disparity between male and female students, with males exhibiting a higher prevalence of non-fatal injuries. This finding aligns with previous research (54, 59, 60). The discrepancy may be due to males being more prone to encountering potential risk factors, as they often engage in sports activities, risk-taking behaviors, social activities, etc. In Chinese culture, boys are often expected to act braver and more assertive, a cultural norm that may contribute to their greater tendency to engage in high-risk activities, such as extreme sports and risk-taking behaviors, thereby increasing the probability of injury. On the other hand, girls are taught to follow safety rules and avoid risky behaviors, allowing them to grow up with relatively little exposure to risk. Research suggests that mothers and fathers may have different approaches to parenting, with mothers preferring protective measures for their daughters and fathers encouraging more risk-taking behaviors for their boys (61, 62). However, further comprehensive research is required for substantiation.

This study found that different socioeconomic status and parenting practices among males and females are associated with the incidence of nonfatal injuries. There is a statistically significant difference in injury rates among students from good, moderate, and poor household financial situations. Pairwise comparisons indicate significant differences between students from moderate financial situations and the other two groups but no significant difference between students from poor and good financial situations. This may reflect the influence of family background on individual behavior and safety (63). Specifically, parental or guardian respect for privacy can lead to a lower incidence of serious injuries, signifying that a more respectful approach to education may enhance adolescent wellbeing (64). Research has shown that high levels of parental monitoring (i.e., parental knowledge of the adolescent’s activities and social circles) are associated with lower rates of risky behaviors such as violence, substance use, and mental health problems (65). Students from higher socioeconomic status invest more time in sports (66), especially in more competitive sports, as shown in a study that showed that the incidence of injuries during training and competition was significantly higher among students from high-family incomes than among students from low-income families during the epidemic (67). Future research could further explore the relationship between economic background and risk behavior to understand this phenomenon better. It can be posited that family financial status affects an individual’s access to safety facilities and resources. Furthermore, parenting practices, including respect for privacy, may result in individual differences in risk and safety awareness.

Although smooth relationships are often thought to provide emotional support and stability, the present study found that this group of students instead faced a higher risk of injury. This may be related to higher levels of social activity among these students. First, research suggests that students in stable relationships may be more involved in shared physical or social activities, often accompanied by some physical risk. For example, couples may engage in outdoor adventures, sports, or other activities that require physical participation, which inherently carries a higher risk of injury. Due to the trust and closeness of the relationship, partners may encourage or challenge each other, leading to a willingness to attempt riskier behaviors that increase the likelihood of injury (68). In addition, individuals in stable relationships may be less alert to risk and less concerned about safe behaviors due to emotional relaxation, increasing the risk of unintentional injury (69).The findings demonstrate a significant association between less internet time (6 h or less per day) and lower rates of non-fatal injuries. This aligns with existing literature suggesting that excessive internet use may lead to a higher incidence of injury (70). Specifically, it can reduce social activities and physical exercise, thus compromising physical health and psychological state (71). For instance, internet addiction is closely linked to other risky behaviors such as a sedentary lifestyle, irregular diet, and sleep debt (72, 73), further increasing the probability of injury. A UK-based longitudinal study found a positive association between time spent on the Internet and subsequent mental health problems, particularly among young people who were online for more than 6 h per day (74). Other studies have noted that excessive Internet use is often associated with problematic Internet use and that this over-reliance on the Internet can lead to a range of mental health problems, such as depression and anxiety (75).

In this study, the chi-square test showed that the injury rate of only children was higher than that of students with siblings, suggesting that only-child status may be an influential factor. However, the SHAP value of Random Forest showed that only-child status may have a protective effect. This contradiction may stem from the difference in analytical methods: the chi-square test focuses on a single variable, while the random forest considers the combined effect of multiple variables. It may be that there may be a complex relationship between only-child status and factors such as family economic status or educational style that affect the risk of injury. Therefore, future research could further explore the combined effects of family factors on student health and safety to provide a more targeted basis for health interventions.

This study found that students with poor physical health had significantly higher injury rates than those with good health, a finding consistent with existing research (76). In addition, college students in poor health not only suffer in their academic performance, but their physical and psychological state also tends to make them more susceptible to injuries in their daily activities. A study of college students noted that students with higher levels of stress and anxiety were in poorer health and were more likely to suffer from conditions such as muscle strains or falls when coping with physical activities (77). This is supported by this study’s finding of a significant relationship between poorer physical health and high injury rates. These findings suggest that schools need to reduce students’ risk of injury through a combination of interventions that enhance physical fitness and mental health support.

In terms of academic pressure, significant differences are observed between high and moderate levels, as well as between moderate and none, but not between high and none. This pattern suggests that moderate levels of academic pressure may uniquely impact students in ways that are distinct from both high and no pressure.

The finding that students in noisy bedroom environments have a higher injury rate than those in quiet environments is supported by recent research linking noise exposure to increased health and safety risks. Cho et al. (78) demonstrated that noise pollution negatively impacts sleep quality and elevates stress levels, impairing cognitive function and increasing susceptibility to accidents and injuries. This suggests that noise in bedroom environments may compromise student safety by affecting their mental and physical wellbeing, highlighting the importance of a quiet living environment for reducing injury risk.

The finding that extroverted university students have a higher injury rate than introverted students is consistent with research suggesting that personality traits can impact safety and risk behaviors. Wen et al. found that extroverted individuals are more likely to engage in social and physical activities that increase their exposure to injury risk factors (79). This suggests that extroverted students may be at a higher risk for injuries due to greater social engagement and risk-taking behaviors, emphasizing the importance of tailored safety interventions based on personality traits.

The finding that risk-taking students have a higher injury rate than non-risk-taking students aligns with recent research on young adults. A study investigated the relationship between risk-taking behaviors and injury rates in university students, finding that those with higher risk preferences reported more injuries due to engaging in high-risk activities (80). This suggests that targeted safety interventions could benefit students inclined toward risk-taking behaviors to reduce their injury risk.

The results of this study showed that the chi-square test showed that high levels of depression were significantly associated with higher injury rates. However, the SHAP analysis of Random Forest showed that depressive status had a smaller effect on injury. The results of this analysis suggest that although the depressive state is a single risk factor, its effect in a multivariate setting may be partially offset by other health and behavioral factors (81). This suggests that unifactorial and multifactorial analyses each have value in assessing injury risk and may complement the understanding of potential health impact mechanisms.

This detailed information allows for a clear understanding of each factor’s specific contribution and influence direction in the prediction of non-fatal injuries. This understanding provides a crucial basis for formulating intervention measures, identifying high-risk demographics, and implementing effective prevention and intervention.

### 4.4 Comparative analysis of machine learning models for predicting non-fatal injuries

In this study, several ML models were utilized to construct classification models, including Random Forest, Decision Tree, Logistic Regression, and XGBoost, each offering a unique perspective on the data. Among them, Random Forest and XGBoost excel at capturing complex data patterns and relationships, whereas Decision Tree provides an easily interpretable structure, and Logistic Regression can identify linear associations. The Random Forest model produced a larger area under the curve (AUC) than the other three methods, indicating its exceptional applicability for research on preventing non-fatal injuries among college students. While cross-validation effectively reduced model overfitting on the training data, we plan to consider in future research other ways model robustness can be further strengthened through specific methods or independent test sets.

## 5 Limitations

This study has several limitations. First, recall bias may occur due to the self-reporting methodology adopted and the 12-month recall period for most research data/information. Second, the cross-sectional design of the study prevents the drawing of causal conclusions. Third, practical constraints hindered the use of external data for model validation. Finally, the study was conducted solely in Hainan, potentially limiting the generalizability of the findings to broader regions. In this study, we focused on analyzing the total number of nonfatal injuries in the college population and did not provide a detailed breakdown of injury types. Future research will further explore the specific risk factors for different injury types for a more detailed analysis. Although we defined nonfatal injuries based on the criteria of a medical diagnosis or leave of absence of more than 1 day, we did not further categorize injury type or severity in this study.

While the results of this study provide important insights for understanding the risk of nonfatal injuries among college students in Hainan Province, we recognize that the generalizability of the results is limited. Future studies could be replicated in other Chinese provinces (e.g., Guangdong, Guangxi, Fujian, etc.) as well as in universities internationally to verify the generalizability of the findings. In addition, researchers could consider different types of universities and student populations to more fully explore injury risk among college students.

## 6 Conclusion

Non-fatal injuries among university students in Hainan Province, China, are a significant public health concern. Early understanding of students’ characteristics and behaviors is crucial for implementing effective interventions, such as health education courses, to prevent potential injuries and related consequences. This study has identified five main factors affecting non-fatal injuries: sex, household financial situation, relationships going well, internet time, and only child. Strategies targeting these risk factors may contribute to the prevention of non-fatal injuries. Additionally, the study compared the strengths and weaknesses of different ML models in this field of research, providing a valuable reference for future research.

## Data Availability

The raw data supporting the conclusions of this article will be made available by the authors, without undue reservation.
